# The Usage of Mobile Apps to Fight Violence against Women: A Survey on a Sample of Female Students Belonging to an Italian University

**DOI:** 10.3390/ijerph18136968

**Published:** 2021-06-29

**Authors:** Pamela Tozzo, Andrea Gabbin, Caterina Politi, Anna Chiara Frigo, Luciana Caenazzo

**Affiliations:** 1 Legal Medicine Unit, Department of Molecular Medicine, University of Padova, 35121 Padova, Italy; luciana.caenazzo@unipd.it; 2 Department of Cardiac, Thoracic, Vascular Sciences and Public Health, University of Padova, 35121 Padova, Italy; andrea.gabbin@gmail.com (A.G.); caterina.p.politi@gmail.com (C.P.); annachiara.frigo@unipd.it (A.C.F.)

**Keywords:** mobile apps, violence against women, survey, personal safety

## Abstract

The prevalence of violence against women continues to grow and this plague has had a huge impact from a clinical, social and judicial point of view. For this reason, alongside the efforts made at the legislative level to prevent the phenomenon and to improve assistance to victims in recent years, efforts to contain and better manage this phenomenon have also grown in the extra-legislative sphere: for example, through the application of new technological solutions and safety planning. In recent years, there has been an increase in the marketing of mobile phone apps dedicated to the prevention of violence against women, with different functions and different objectives. The purpose of this study is to investigate the knowledge and propensity to download this type of app in a group of 1782 Italian female university students. This research was performed using an online questionnaire administered to female students attending four different courses (law, medicine, healthcare professionals and political sciences) at one Italian university. Chi-square or Fisher’s exact test was used to analyze associations between responses to questionnaire and the type and the year of course. The results show that 62.6% of our sample are unaware of the existence of these apps and that 79.5% of the sample would be willing to download one in the future. With regard to whom to turn to after a violent incident, the majority of those interviewed (43.9%) would turn to the police and not to health facilities. According to our findings, law female students (52.7%) think, more than any other category, that the most effective way to improve public safety and reduce the number of victims lies in legislative solutions. Our results suggest that, although this type of technology may be promising, it is necessary to improve the knowledge and dissemination of these apps in order to make them a useful tool for prevention, education and assistance in cases of violence against women.

## 1. Introduction

The United Nations defines violence against women as “any act of gender-based violence that results in, or is likely to result in, physical, sexual, or mental harm or suffering to women, including threats of such acts, coercion or arbitrary deprivation of liberty, whether occurring in public or in private life.” [1]. According to the latest available data provided by the World Health Organization (WHO), globally about 1 in 3 (35.6%) women worldwide have been subjected to either physical and/or sexual intimate-partner violence or non-partner sexual violence in their lifetime [2]. The term “violence against women” encompasses different forms of violence, including violence perpetrated by an intimate partner (intimate-partner violence) and rape/sexual assault or other forms of sexual violence perpetrated by someone other than a partner (non-partner sexual violence), as well as female genital mutilation, honor killings and the trafficking of women.

Based on the Italian National Institute of Statistics (Istat) data relating to years 2014/2015, 6,788,000 women in Italy have suffered some form of physical or sexual violence in the course of their lives and 3,466,000 are victims of stalking [3]. The spread of the phenomenon of violence against women continues to grow and this plague has highly significant consequences from a clinical, social and judicial point of view. For this reason, alongside the efforts made at the legislative level to prevent the phenomenon and to improve assistance to victims in recent years, efforts to contain and better manage this phenomenon have also grown in the extra-legislative sphere, for example through the application of new technological solutions and safety planning [4]. It is starting from this assumption that the phenomenon of the spread of apps dedicated to female (potential) victims of violence was born and grew [5,6,7].

Smartphones, in particular, may in this sense become security allies thanks to the use of some applications that help to make any request for help and/or sending an alarm signal quicker, easier and more effective [8]. Against gender violence the Italian legislation puts forth three objectives: to prevent crimes, to punish perpetrators and to protect victims. As part of preventive measures, Italian legislation has spread the creation of anti-violence centers, has established a toll-free number (1522), and adopted common protocols in healthcare facilities and models of specialization in law courts. To ensure prompt identification of cases of violence and avoid any form of blaming the victim (the so-called secondary victimization), a series of protocols has been developed with which health and law-enforcement operators can handle adequately as well as standardized interventions related to gender-based violence at every stage, from healthcare to the courtroom.

The situations in which a personal safety app can come in handy are more numerous than one can imagine and these apps can help protect citizens against violence and become a “virtual bodyguard” that can intervene when necessary [6]. Since 2014, women have had a silent ally against gender-based violence such as anti-violence apps for mobile devices [9]. While some focus on prevention and information through tests and listening desks, others offer emergency expedients in the context of aggression and emergency numbers, many are able to locate the nearest anti-violence centers, in order to receive concrete support as soon as possible [10]. Based on the nature of the service provided, these apps can essentially be of three types.

Information apps: this group includes those apps which, although they provide useful and emergency telephone numbers, they have not been conceived as an emergency response, but rather as an application dedicated to the development of awareness of certain issues and the prevention of situations of violence or of danger;

Tracking apps: these apps are used to provide someone with their geolocation, so that the “virtual guardian” can intervene if a request for help is sent. These are apps that are useful not only in the event of actual danger, but also to reassure a relative, friend or partner when traveling, when going for a hike alone or walking on the street at night;

Emergency apps: the last category includes the applications to be used when the user thinks to be in a situation of perceived or real danger and she needs the immediate support of someone, even just by telephone and not necessarily from the police. These apps, therefore, allow for the launching of a quick and effective alarm in which, even in a crisis situation, the victim’s position can automatically be sent.

Hundreds of apps of this type are currently available on the international and national market and can be easily downloaded and used. The functions that these tools for women’s safety provide can be summarized as follows.

Behavioral advice: some apps perform an educational and informative function, including useful advice for women of all ages. These are good practices to prevent dangerous situations or to know how to deal with them in the best possible way;

Legal information and support centers: some apps dedicated to the prevention of violence against women include information sections on the most important legislative aspects, together with practical information on the listening or anti-violence centers that can be found in the region of residence;

Alarms and sounds: many applications include buttons that activate signals (alarm sounds, a fixed or intermittent flashlight, etc.) that are useful for attracting the attention of people in the vicinity and disorienting or, even better, warding off any possible perpetrator;

Emergency calls: in some cases, it is sufficient to press a button to directly call emergency numbers;

Company and virtual support: the most advanced programs also include a real call center active 24 h a day and every day of the year, so as to be able to provide telephone company for the user who does not feel comfortable in a particular situation;

Tracking and sending position: many applications act as a tool for monitoring your position, using the GPS and thus allowing you to geo-locate the user and send the position to rescue or to some pre-set contacts in the event of an emergency;

Community: in some cases, it is possible to report the presence of any dangers to all other users of the same app who are nearby, so that they can avoid the area, or cross it with particular attention;

Insurance in case of damage: among the various available apps analyzed we have identified one that stands out because it does not only deal with safety in terms of prevention, but also with remedies. In fact, it provides insurance that covers medical assistance, lock restoration and key or document restoring.

Other very important aspects are the way in which the app presents itself to the user, along with the design choices that have been made by those who created it and the simplicity with which all information and functions are accessible, especially by women of different ages. We should bear in mind, in fact, that these applications can be used by women of any age, and it is, therefore, essential that they are accessible quickly and intuitively by any type of user, regardless of the familiarity that one has with technological tools.

Most of the applications analyzed are free, while those that are not free may have a variable cost: for some, an activation fee must be paid, while others have an annual fee which, if desired, can be paid in a lump sum or monthly. In the group of paid apps, however, the most complete ones are included, which may provide additional services for the user and the prices are usually not staggering—ranging from about EUR 20 to a maximum of EUR 40 per year.

Even if from 2016 to 2020, 218 billion apps have been downloaded, security apps seem not to be among the most downloaded ones [11] and few studies have analyzed the real usage rate and the perception of users of anti-violence apps [12]. Glass et al. in 2015 published a randomized controlled trial testing the effectiveness of a web-based, interactive safety decision aid and smartphone application for abused college women and their friends and the authors highlighted the importance of expanding studies on the efficacy and use of apps for safety by young women [13]. McCarthy et al. in 2016 performed a survey on aspects of personal safety apps and how transport users perceived them in relation to their personal safety, privacy and their preference to purchase them [14]. The majority of respondents would consider downloading a personal safety app, and the authors suggested this technology being worth pursuing and that further research concerning the use of personal safety apps would be interesting. More recently, Potter et al. examined a sample of community college students who reported downloading a violence prevention and response mobile app and their reasons for downloading it; the author concluded that a mobile app may be a promising mechanism to provide sexual violence prevention and response information to college-age populations that are difficult to reach [15].

Thus, starting from the interest expressed in the literature for further studies on the use of anti-violence apps and on the usefulness of investigating the opinions of young people, the present article aims to understand the knowledge and usage of violence-prevention-and-response apps in a sample of female Italian university students. The study, in particular, was conducted on a sample of female students enrolled in a single university located in the north east of the country (University of Padua), restricting the target to four degree courses with a high number of students and a large female component. Degree courses with different training objectives were involved, in order to explore if and what the impact of the training of each course (with respect to the contents) was on the creation of a sensitivity towards this type of problem and the knowledge of how mobile apps can prevent or combat the phenomenon.

As far as we know, this is the first study conducted in Italy to investigate the perception of a sample of female students belonging to a single university regarding the availability and effective use of this type of application for mobile devices.

## 2. Materials and Methods

Once we established the general aims of the study, a questionnaire was developed by the research team, using a similar structure that was previously used in another study involving students belonging to the same university [16,17].

We decided to structure a questionnaire of 14 questions, so that it could be easily and quickly filled out, which, first of all, investigated the type of course and the year of the course and, subsequently, some general aspects on violence against women and on the use of app to prevent and manage this phenomenon. We chose the questions on the basis of other works in the literature, analyzing in particular the theme of privacy which in previous works, both of our group [16,17] and of other groups [14,15], seemed to be very much felt by the respondents.

**Figure 1:**
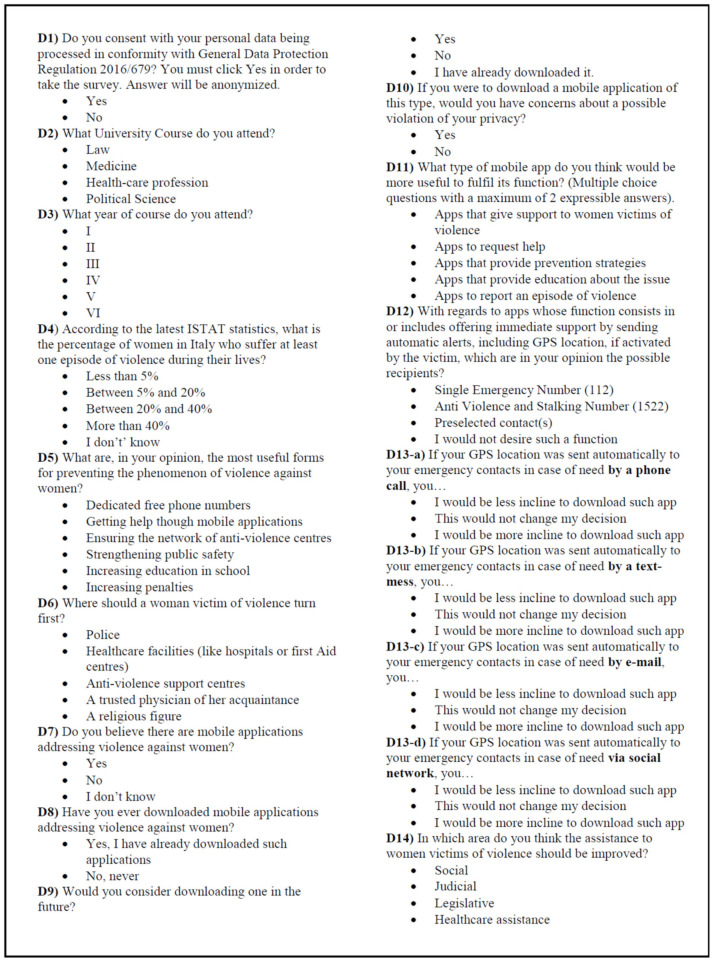
English translation of the administered questionnaire.

Figure 1 contains the English translation of the questionnaire.

The questionnaire was subsequently tested on a small number (*n* = 30) of young women (19–30 years old) belonging to different professional areas at our institution, reflective of potential respondents. On the basis of their comments and suggestions, questions were modified, with the help of biomedical statistical expert. In particular, following the suggestions provided, for question D11 we foresaw the possibility of giving more than one answer and we added section d to question D13, i.e., the possibility of reporting violence to an emergency contact through social networks.

The final version of the questionnaire was then submitted during one academic semester in 2020 to female students enrolled in the four chosen degree courses. The responses of 1782 female students were analyzed, and a statistical analysis was conducted on the resulting data.

Inclusion criteria for participation in the survey were female gender, use of smart phones, understanding the contents of apps in smart phones and current enrolment at university courses. All questionnaires were filled out anonymously. The questionnaire was drafted in Italian and distributed as a link to Google Forms^®^ using the institutional mailing lists of the female students attending four different courses: law, medicine, healthcare professionals (nurses, physiotherapists, speech therapist, occupational therapists and midwifes) and political sciences. We decided to ask the type of course and the year of the course, assuming that these two pieces information could influence the answers of the students, for example due to a greater propensity of law/political sciences students to highlight the judicial strategies for preventing the phenomenon, while medicine/healthcare professionals may be more sensitive to the victim’s healthcare aspects. Respondents were asked whether they were aware of the prevalence of violence against women in our country, if they were aware of the existence of mobile phone apps that aim to fight or prevent violence against women, their level of confidence in these technologies, and whether they would consider using these technologies. In order to obtain a better understanding of young female students’ attitudes towards mobile apps against violence, participants were asked about which types of apps could be considered most useful and how their opinion on these apps could change according to the subjects alerted by the app in case of need. All questions were mandatory; therefore, it was not possible to send incomplete questionnaires. We then considered the 1782 questionnaires sent, which were all complete.

Data were registered in an Excel file for statistical analysis using SAS 9.4 (SAS Institute Inc., Cary, NC, USA) for Windows. Results are presented as number and percentage of respondents for each option of the questions.

Chi-square or Fisher’s exact test was used to analyze associations between responses to questionnaire and the type and the year of course. The statistical significance was declared when *p* < 0.05.

A specific authorization from the competent ethics committee was not necessary, and the ethics committee requested to communicate our intent to the programme coordinators of the courses concerned and ask them for permission to submit the questionnaire to the students. All interested programme coordinators expressed their favorable opinion.

## 3. Results

A total of 1782 responses to the online survey were recorded, out of which 567 were received from law students, 546 from medical students, 544 from healthcare professional students and 125 from political science students. Distribution of type and year of degree is given in Table 1.

Concerning general awareness of the prevalence of violence against women (question D4) (according to the latest Istat statistics, what is the percentage of women in Italy who suffer at least one episode of violence in the course of their lives?), answers were distributed as follows: 37.6% of voters think that between 20% and 40% of Italian women have been victims of at least one episode of violence, 30.2% think the prevalence rate exceeds 40%, while only a minority of respondents (7.8%) believes it is lower than 20% (7.7% answered “between 5 and 20%”, and 0.1% said “less than 5%”). Finally, 24.3% had no idea as to the correct answer.

The most effective prevention strategies having to do with violence against women are thought to be (question D5) (*What are, in your opinion, the most useful forms for preventing the phenomenon of violence against women?*): furthering gender equality and women’s rights through education in school (81.1% of voters), strengthening public safety (52.8%), ensuring the network of anti-violence centers (48.0%), increasing penalties (47.9%), and getting help through dedicated free phone numbers (34.4%) or mobile applications (27.2%).

In order to get support in case of violence (question D6) (*Where should a woman victim of violence turn first?*), 43.9% of respondents would first turn to the police, 26.4% to healthcare facilities (like hospitals or first-aid centers), 25.3% to anti-violence support centers, 4.3% to a trusted physician of her acquaintance, and only one respondent (0.1%) would rather consult a religious figure.

With regard to the existence of mobile applications specifically designed to assist women who are experiencing violence and help them to escape abuse (anti-violence apps), only 33.1% of respondents are aware of this opportunity, while the great majority of voters (62.6%) professed “I don’t know” (question D7) (*Do you believe there are mobile applications addressing violence against women?*).

Furthermore, almost all respondents (98.8%), including those who are aware of their existence, declare they have never downloaded such an app in their mobile phone (question D8) (*Have you ever downloaded mobile applications addressing violence against women?*).

This survey found that most participants would consider downloading an anti-violence app in the future (79.5%), and only a minority do rule out a priori this possibility (19.8%) (question D9) (*Would you consider downloading one in the future?*).

The association between the knowledge of the extent of violence against women, the awareness of the existence of these apps and the aptitude to download one is described in Table 2. The percentage of women who are unaware of the existence of these apps is higher among those who do not know how many women have suffered at least one episode of violence. Furthermore, the percentage of those who would download the app is higher among the respondents who believe that the percentage of women who have suffered violence is greater than 40%.

When asked what type of mobile app they think would be more useful to fulfil its function, 85.2% of voters replied “app to request help”, 62.7% “app to report an episode of violence”, 25.6% “app that gives support to women victims of violence”, 17.1% “apps that provide prevention strategies”, and 9.7% “apps that provide education about the issue” (multiple choice question D11).

With regard to apps whose function consists of or includes offering immediate support by sending automatic alerts, including GPS location, if activated by the victim (so-called “emergency app”) [10], possible recipients, according to our interview, should be the Single Emergency Number (112) (56.0%), the Anti-Violence-and-Stalking Number (1522) (39.7%), or (a) preselected contact(s) of the victim (3.5%). In total, 0.7% of respondents would not desire such a function (question D12).

Most of the voters stated that they would be more inclined to download the app if the help request was sent through a phone call (59.9%) or a text message (56.7%), while if it was sent via social network, then they would be less inclined to download it (47.1%). A help request sent by e-mail does not affect responders’ choice between downloading the app or not (45.6%) (question D13) (*If your GPS location was sent automatically to your emergency contacts in case of need, you* …).

Finally, responses to question D14 (In which area do you think the assistance to women victims of violence should be improved?) indicate the need for improvement mainly in the social area (45.5%), followed by judicial (36.1%), legislative (15.9%) and, lastly, healthcare assistance area (2.5%).

With regard to preventive measures, medical students (86.3%) find that school-based education represents the most important intervention in fighting violence against women, especially in comparison to law (77.4%, adjusted *p*-value 0.0008) and healthcare professions (79.4%, adjusted *p*-value 0.0162). Law students (52.7%), on the contrary, believe that increasing punishments will be more effective—especially compared to medical students (43.4%, adjusted *p*-value 0.0111)—but with a declining trend as the year of course increases: this answer choice was higher among students attending the first year of course and lower among those attending the last (fifth) year, with *p*-value of 0.0022.

Similarly, there exist statistically significant differences concerning to whom the woman victim should turn first in the case of violence. Although the most common answer, regardless of course type and year, was “law enforcement”, law students seem more convinced of this option (51.7%) than the others, especially medicine students (37.4%) (*p*-value < 0.0001). Considering the same question, students in the healthcare sector, both medicine and healthcare professionals, expressed a higher preference for the “first-aid/hospital” option (respectively, 28.6% and 30.7%) compared to law and political science students (21.3% law and 21.6% political sciences).

According to this survey, there is an inverse relationship between this question and the year of course: the percentage of medical students who answered “police” is higher among those in their first three years of school (respectively, 38.0%, 42.7%, and 44.3% in the first, second, and third year), and progressively decreases in the remaining three course years (*p*-value 0.0004). At the same time, and in parallel, healthcare entities (including both “first-aid/hospital facilities” and “family doctor”) become of increasing importance as a point of reference among students in their last three course years, up to a maximum of 51.8% in the sixth year. A similar trend (*p*-value 0.0035) was also observed among law students: the “police” answer was given by 57.2% of students in the first year versus 37.3% in the last (fifth) year, with a concurrent increasing in “first-aid/hospitals” answer, from 13.9% up to 35.6%.

In addition, we found that the use of apps addressing violence against women, including location data, raise privacy concerns more among law (34.2%) and healthcare professional (36.4%) students compared to medical (25.6%) students (law adjusted *p*-value 0.0108; healthcare professions adjusted *p*-value 0.0007).

A priority area for improvement has been identified by respondents in the social sphere, with a maximum among medical students (50.2%) and a minimum among healthcare professional students (40.3%) (adjusted *p*-value 0.0132). Very few students (from 1.6% of law and medicine to 4.0% of healthcare professionals) believe the medical/assistance sector requires priority improvement in the management and prevention of the phenomenon.

Among healthcare professional students’ preferences, we observed a statistically significant trend depending on the year of course in three questions (D5, D9, and D11). First, education in school is seen as the most useful tool in preventing violence against women with a growing trend which is directly proportional to the year of the course, with a minimum value in the first year (73.1%) and a maximum value in the third year (84.2%) (*p*-value 0.0187). Second, in question D9 (*Would you consider downloading one in the future?*) the percentage of affirmative answers decreases in an inversely proportional way to the year of the course, from a maximum in the first year (82.6%), down to a minimum value in the third year (72.1%) (*p*-value 0.046). Third, an increasing trend was observed with regard to what type of mobile apps addressing women victims of violence is considered more useful (“app for support”: 16.4% among students in their first course year versus 27.3% of the third (last) year (*p*-value 0.0348)).

Among political science students, only the question (“educational app” in question D11, concerning the most useful kind of app) showed a statistically significant increase depending on the year of course, with a minimum value in the first year (16%) and a maximum value in the third year (20%) (*p*-value 0.0129).

Again, those believing the phenomenon of violence against women is fairly widespread (“20–40” or “above 40%” of all women) are more aware of the existence of dedicated apps (respectively, 33.9% and 37.5%) (*p*-value 0.0123), compared to those thinking the prevalence rate of the phenomenon is lower than 20% (31.7%) or who do not know its exact prevalence (26.9%). On the other hand, students with less perception (<20%) or scarce knowledge (“do not know”) of the magnitude of the phenomenon are in parallel less conscious of the existence of anti-violence apps.

A similar trend is observed among answers to question D4, concerning the extent of the phenomenon (“>40%”, “20–40%”, “<20%”), and the eventuality of downloading an anti-violence app (affirmative answer in the 83.3%, 78.5% and 75.5%, respectively).

As might be expected, apps addressing violence against women have been downloaded more frequently by students who already know about (or believe in) their existence rather than by students who are unaware (3.7% versus 0.0%) (*p*-value < 0.0001). On the contrary, the latter would take into greater account the idea of downloading one in the future (83.1% versus 78.8%) (*p*-value < 0.0001).

Finally, a statistically significant association is observed between positive versus negative predisposition towards downloading such an app and privacy concerns (*p*-value < 0.0001): 70.2% of students who would consider downloading one have no concerns about a possible violation of their privacy, while amongst students who would not consider such an idea the rate is significantly lower (58.5%) as reported in Table 3.

## 4. Discussion

Technology already plays, but will play even more in the near future, an extremely important part in the process of improving community life. After all, it is now clear to everyone, how going digital has become fundamental for the implementation of projects in support of strategic fronts, from health to education, from communication to security. Mobile phones are an integral part of our life and influence many aspects of our social and personal life. In Italy, in 2019, among users aged 14 and over, 91.8% used smartphones to navigate the internet. The use of mobile apps is a daily activity for most individuals and the development of these technologies is rapid and growing, to the point that it is difficult to offer a synthetic picture of all the new technologies available. These are technologies that promote strong innovation and open new opportunities with respect to the protection of health, the promotion of healthy lifestyles and, last but not least, the implementation of citizens’ safety.

According to the latest ISTAT data, 31.5% of women in Italy have suffered at least one episode of violence in their lifetime. In our survey, little more than one third of respondents (37.6%) gave the correct answer, thus suggesting that female students belonging to our university, despite their high educational level and regardless of their field of study (both healthcare and legislative/administrative sector), have a limited knowledge of the real extent of the problem. This data is confirmed by the high percentage of respondents admitting they did not know the answer (24.4%). The reason for this lack of knowledge should be investigated further in order to understand whether it is to be attributed to an underestimation of the problem or to a lack of interest on the part of the interviewees, given that the press is still very active and very punctual in reporting the statistics and news episodes concerning violence against women. However, distribution of incorrect answers shows that our respondents have a tendency to overestimate (30.2%) the prevalence of the phenomenon, rather than underestimate it (8.7%).

The data in this questionnaire show that female students have little knowledge of the availability of anti-violence apps (only 62.6% of respondents know of the existence of these apps) and 98.8% say they have never downloaded one. Surprisingly, although the sample interviewed concerns young and well-educated women, only one third of them are aware of the availability of these apps on the market, and practically all interviewees declare that they have never downloaded one. Although referring to a different target population, McCarthy and collaborators found similar results in their study: in fact, 82.4% of the interviewees were not aware of the safety apps and 62.9% were interested in downloading one [16]. Similarly, Potter et al. reported that in their study, although referred to the knowledge of a specific app (uSafeUS), only 20.0% of participants had heard about it and its features [15].

Furthermore, our data is partially congruent with what has been highlighted by Statista in 2019 on the digital skills of Italians. In fact, according to these data, those who are still in the education system use the Internet more and better, but not enough. The percentage of young people who do not have at least basic digital skills (the minimum ones to exercise citizenship rights) is still high and reaches 30%. It should also be considered that, compared to 98.8% of respondents who said they had never downloaded these apps, 79.5% said they would consider downloading one in the future. This could lead us to think that there is no effective way of advertising these apps. Therefore, marketing policies and incentives for the use of such apps could be encouraged. Since user involvement has been shown to be one of the most useful factors for app programming, and given the low awareness shown by our sample of women, it could be useful to develop new studies to understand the needs and expectations of women with respect to these apps [18], as suggested and demonstrated for other types of apps [19,20,21]. App programmers should take into account the results of studies of this type and implement the advertising and dissemination channels of apps for security.

We found a statistically significant association between positive versus negative predisposition towards downloading such an app and privacy concerns: in particular, the majority (70.2%) of respondents who would consider downloading one have no concerns about a possible violation of their privacy, while amongst students who would not consider such an idea the rate is significantly lower (58.5%), similarly to what has been reported by McCarthy et al., who found that the majority of respondents (69.3%) reported that they would have no concerns for their privacy when using a personal safety app [14].

The goal of improving the knowledge and use of this type of app by women could be achieved through various strategies: encouraging developers and industries to produce apps that are actually useful and reliable for the safety of citizens; the establishment of monitoring observers of the apps with regard to the protection of security in view of the identification of potential risks; the establishment of websites and/or accredited portals with indications on the classification of the available apps constantly updated, in relation to the functions and any risks; the monitoring and adequate education of particularly vulnerable categories in order to guarantee non-discrimination and encourage inclusion from the advantages of the use of new technologies. It is therefore evident that among the forms indicated as most useful in the prevention of violence against women, the use of dedicated apps was chosen by the minority of respondents (27.2%).

With regard to the figures to turn to in case of need after an episode of violence, the majority of those interviewed (43.9%) would turn to the police and not to health facilities. This data suggests that the aspects related to the healthcare of women victims of violence are considered secondary in our country, despite the efforts made by the legislator to create dedicated paths and shared protocols of assistance in hospitals for these victims.

According to our findings, law students (52.7%) would think, more than any other category, that the most effective way to improve public safety and reduce the number of victims lies in legislative solutions, i.e., increasing the severity of punishment for the perpetrator(s). On the contrary, the great majority of medical students (86.3%) believe the most important initiatives to prevent violence against women lie in higher education and positive social changes. Therefore, it seems that the field of studies influences the perception of the violence prevention strategies of the interviewed students.

Moreover, the sample we interviewed is highly confident in the educational role of the school as the main strategy for the prevention of violence against women. There seems to be a direct relation between the perceived severity and the spread of the phenomenon of violence against women, and the degree of knowledge of apps specifically created to address such situations.

According to our results, the reporting of the dangerous situation through social networks would discourage the downloading of these apps and, therefore, despite the fact that social networks are widely used by young people, the respondents would prefer that the alarm signal be sent through more traditional channels, such as a phone call or a text message.

The sample analyzed in this survey is made up of young university students belonging to a single university located at the north east of Italy, whose high educational level and cultural resources are adequate to be able to understand the usefulness of these applications. In analyzing our results, however, it should be borne in mind that the Italian female population is highly heterogeneous and many women could be excluded from accessing these technologies. To the extent that the undoubted advantages of these technologies in the service of security are recognized, the problem of the exclusion of those who do not have access to technology emerges, i.e., the gap between those who have tools and skills and those who are marginalized due to lack of technology or lack of knowledge of the use of technology. In this sense, it is essential to reflect on the expansion of the right of access to new technologies for all, including disadvantaged and particularly vulnerable groups, in the context of a just distribution of technological resources.

Taking into account the results of this survey, which show the lack of awareness of our sample respondents about the possibility of downloading and using this type of app, an indispensable element is the appropriate education of citizens in the use of new mobile security technologies, in order to acquire adequate skills for the appropriate use of these tools. In addition, non-discrimination must be guaranteed for those who will not be able or will not be able to access these technologies, obviously always guaranteeing the availability of alternative solutions for the protection of the safety of citizens and in particular of the most vulnerable groups.

Moreover, we should not forget the promotion of studies on the impact of the use of apps, such as the one we have conducted, with specific attention to the implications on personal and relational identity, in order to clearly identify the problems of addiction and technological vulnerability.

The major strength of this study is the large number of subjects who answered the questionnaire. Although the results cannot be considered representative of the entire Italian university female student population, to our knowledge this survey is the first survey performed on an Italian university focusing on anti-violence mobile app use among female students. In particular, we examined different subgroups of students belonging to different courses (law, political sciences, healthcare professionals, medicine and surgery) in which there is a high representation of female students. Moreover, considering the type of questionnaire, participants had the opportunity to look into their devices while answering the online questions, to gain more awareness of these technological devices.

A limitation of this study is that it was conducted in a single university and on a limited number (four) of study courses. Thus, the results found in this study need to be replicated in longitudinal and multi-centric studies. Nonetheless, as these apps are commonly available, and will probably be widespread in the near future, it would be useful and valuable to investigate further women’s awareness, knowledge, and predisposition to use these types of mobile technologies. Further limitations include the collection of data relating to the usefulness perceived by women rather than data obtained from objective measures of apps’ actual ability to prevent violence and the choice of involving a sample of undergraduate women at a single public university, thus limiting the generalizability of our results to other populations.

## 5. Conclusions

While this study is qualitative and limited to a single Italian university, our findings suggest some important aspects on the knowledge of a sample of female university students regarding anti-violence apps. The importance of our findings highlights that these apps are not known, nor are they used, precisely by the target audience of women that we interviewed. Nevertheless, it is interesting to note that many participants would be interested in downloading one of these apps. Therefore, it would be advisable for app developers to look for better advertising strategies for these apps and to be known by as many women as possible, so that women can be motivated to download these apps that could be used to help themselves. Certainly, the diffusion of these apps cannot replace other channels of prevention and support for victims, but it can certainly be a useful resource, in particular on the educational and social side, rather than guaranteeing immediate assistance in the event of aggression.

## Figures and Tables

**Table 1:** Distribution of type and year of degree among the 1782 respondents. The legal number of course years is six years for Medicine and Surgery, five years for Law, three years for Political Science and Healthcare Professions.

University Course				
*N* = 1782	Law (*N* = 567)	Medicine and Surgery (*N* = 546)	Healthcare Professions (*N* = 544)	Political Science (*N* = 125)
D3. Year of Course				
1°	194 (34.2)	108 (19.8)	201 (36.9)	75 (60.0)
2°	79 (13.9)	131 (24.0)	160 (29.4)	35 (28.0)
3°	89 (15.7)	106 (19.4)	175 (32.2)	10 (8.0)
4°	62 (10.9)	45 (8.2)	0 (0.0)	3 (2.4)
5°	84 (14.8)	48 (8.8)	8 (1.5)	2 (1.6)
6°	59 (10.4)	108 (19.8)		

**Table 2:** Association between the knowledge of the extent of the phenomenon of violence against women, the awareness of the existence of these apps and the aptitude to download one.

	D4. According to the Latest ISTAT Statistics, What Is the Percentage of Women in Italy Who Suffer at Least One Episode of Violence during Their Lives?				
*N* = 1782	I Don’t Know (*N* = 435)	< 20% (*N* = 139)	20% < 40% (*N* = 670)	4 > 40% (*N* = 538)	*p*-Value
D7. Do you believe there are mobile applications addressing violence against women?					
I don’t know	300 (69.0%)	89 (64.0%)	419 (62.5%)	307 (57.1%)	0.0123
No	18 (4.1%)	6 (4.3%)	24 (3.6%)	29 (5.4%)	
Yes	117 (26.9%)	44 (31.7%)	227 (33.9%)	202 (37.5%)	
D8. Have you ever downloaded mobile applications addressing violence against women?					
No, never	431 (99.1%)	137 (98.6%)	666 (99.4%)	526 (97.8%)	0.0715
Yes, I have already downloaded such applications	4 (0.9%)	2 (1.4%)	4 (0.6%)	12 (2.2%)	
D9. Would you consider downloading one in the future?					
I have already downloaded it	3 (0.7%)	0 (0.0%)	2 (0.3%)	8 (1.5%)	
No	94 (21.6%)	34 (24.5%)	142 (21.2%)	82 (15.2%)	0.0097
Yes	338 (77.7%)	105 (75.5%)	526 (78.5%)	448 (83.3%)	

**Table 3:** Association between the predisposition to download an app against violence against women and concerns in terms of privacy.

	D9. Would You Consider Downloading One in the Future?			
*N* = 1782	I have already downloaded it (*N* = 13)	No (*N* = 352)	Yes (*N* = 1417)	*p*-value
D10. If you were to download a mobile application of this type, would you have concerns about a possible violation of your privacy?				
No	11 (84.6%)	206 (58.5%)	995 (70.2%)	<0.0001
Yes	2 (15.4%)	146 (41.5%)	422 (29.8%)	

## Data Availability

Data are available upon request to the corresponding author.
